# Perceived changes in capability during the COVID-19 pandemic: A Swedish cross-sectional study from June 2020

**DOI:** 10.1177/14034948211023633

**Published:** 2021-07-02

**Authors:** Kaspar Walter Meili, Håkan Jonsson, Lars Lindholm, Anna Månsdotter

**Affiliations:** 1 Department of Epidemiology and Global Health, Umeå University, Sweden; 2 Public Health Agency of Sweden, Department of Living Conditions and Lifestyles, Sweden

**Keywords:** Quality of life, capability approach, COVID-19, cross-sectional survey

## Abstract

**Aims:**

Measures against COVID-19 potentially impact quality of life in different ways. The capability approach by Amartya Sen with a broad and consistent framework for measuring quality of life is suited to capture the various consequences. We aimed to examine (a) whether individuals experienced change in 10 capability dimensions during the first half of 2020, (b) which dimensions were affected most, and (c) whether changes were unequally distributed in terms of gender, education, income, geography, housing, living situation and place of birth.

**Methods:**

We assessed self-reported capability change in Sweden in 10 capability dimensions in a cross-sectional online survey among 500 participants on a five-item Likert scale. We analysed the distribution of answers by comparing the balance of positive and negative perceived changes and used mixed effects logistic regression to examine associations with background characteristics of the participants.

**Results:**

Reported perceived negative changes outweighed positive changes, and a higher proportion stated negative perceived changes if they also stated having low capability in the same dimension. In the capabilities of financial situation, political resources and health, the proportions of perceived negative change were highest. Odds for perceived negative change compared to no or positive change were higher for lower incomes, living in smaller municipalities compared to living in medium-sized municipalities, being born outside Europe, living in the south of Sweden, and renting instead of owning housing.

**Conclusions:**

Self-reported negative capability change, and associated inequalities related to socioeconomic position, place of birth and regional residence should be of concern for policymakers.

## Background

### The COVID-19 pandemic

COVID-19, classified by the WHO as a pandemic on 11 March 2020 [1], is a danger to society, and worldwide preventive measures have been implemented to limit the spread. During the spring of 2020, Sweden implemented a variety of preventative measures linked to elderly care, healthcare settings, restaurants, sporting events, theatres and concerts, national and international travel, education and more [2,3].

The measures against COVID-19 impact societal structures and processes and, in turn, potentially quality of life [4,5]. Countries reported, for instance, decreased gross national products and increased unemployment [6]. Additionally, the indirect consequences may affect the population unequally. For example, vulnerable groups in terms of economic resources and discriminating structures may be more negatively affected than privileged groups. Moreover, public health and the Sustainable Development Goals (SDGs) may be challenged by the COVID-19 pandemic, such as those of no poverty, good health and well-being, quality education, gender equality and decent work and economic growth [7].

The capability approach by Amartya Sen [8] offers a broad and consistent framework for measuring quality of life that can accommodate the diverse effects related to the pandemic [9]. Capabilities refer to the opportunity to achieve a flourishing life according to one’s own wishes. The capability approach measures quality of life in terms of opportunities that people have, in comparison to traditional welfarist approaches that measure well-being in terms of individual utility. The capability approach has been operationalized in various forms [10] and is for example used to assess patient and population health [11], or in clinical trials [12]. For the purpose of this study, we use a list of 10 capabilities published in a public investigation commissioned by the Swedish government [13].

1. Time balance: experiencing a balance between necessary activities (work, other duties) and voluntary activities (social contacts, physical activity, entertainment, hobbies, etc.).
2. Financial situation: having monetary means (salary, other income, or savings) that permit an enjoyable living standard.
3. Health: having a health status (mental and physical) that does not limit the possibility of working or engaging in other desired activities.
4. Political resources: having the opportunity to form an opinion on minor and major issues and being able to present them while being treated with respect (polling stations, associations, authorities, media, etc.).
5. Knowledge: having the education and experience required to work with or engage in interesting issues and purposeful activities.
6. Living environment: being in an enjoyable and practical context (neighbours, buildings, parks/nature, transportation, stores, etc.).
7. Occupation: having satisfying work or other engagements (studies, internships, household work, care of relatives, etc.).
8. Social relations: having access to close relationships (family, friends, acquaintances) that contribute to pleasure and development and give advice and support when needed.
9. Security: not feeling afraid of being afflicted by burglary, vandalism, or various kinds of threats of violence, either at home or in other places.
10. Housing: having affordable and stable housing that is appreciated in terms of functionality, appearance and location.

To our knowledge, no study has assessed capability changes related to the COVID-19 pandemic in the Swedish setting.

## Aims

We aimed to examine (a) whether individuals experienced change in 10 capability dimensions during the first half of 2020, (b) which dimensions were affected most, and (c) whether changes were unequally distributed in terms of gender, education, income, geography, housing, living situation and place of birth.

## Methods

### Participants

We used a commercial web panel for online surveys [14] with stratified sampling for gender (man, woman), education (no schooling, secondary school, high school/tertiary/technical college, university/higher education, postgraduate education), age group (18–22, 23–35, 36–55, 56–80 years) and region of residence (East Middle Sweden, Middle Norrland, Upper Norrland, Stockholm, South Sweden, West Sweden, Småland and the islands, North Middle Sweden), with a target sample size of 500. Stratification proportions were chosen to represent the Swedish population structure, except for education where we chose panel proportions due to difficulties in mapping the panel’s self-reported education categories to categories by Statistics Sweden (Supplemental Section 6). Stratified sampling was applied to complete answers only (after screening out participants declining consent or rejecting the panel’s invitation). The panel-reported response rate for responding to the invitation was 40.96%. Data were collected from 22 June to 6 July 2020. We used Limesurvey [15] hosted on an Umeå University server and registered 560 unique and valid visits corresponding to accepted invitations. Of these, 523 consented to participate. In total 503 answer sets by consenting participants were completed. We excluded three with stated age below 18 (ethical concerns) and two with age above 99 (data quality concerns), resulting in 498 answers.

### Questionnaire

First, we presented information about the study and required participants to give consent to participate. The second section asked participants to indicate their current self-experienced levels for the 10 capability statements (‘agree completely’, ‘agree partially’, or ‘not at all’). In the following section, participants stated perceived capability changes during 2020 for the 10 dimensions on a five-item Likert scale (‘much less’, ‘less’, ‘unchanged’, ‘higher’, ‘much higher’). We avoided mentioning COVID-19 explicitly to prevent potential negative connotations. The survey ended with sociodemographic background questions (Table I; see Supplemental Sections 1 and 2 for screenshots of the survey and English translations). The Swedish Ethical Review Authority approved the study with an advisory statement (Dnr 2019-02848).

**Table I.** Participants’ characteristics and stratification.

Variable	Value	n (%)	Strata	n (%)
Gender	Man	242 (48.6)	No further stratification	
	None of these	7 (1.41)		
	Woman	245 (49.2)		
	NA	4 (0.803)		
Age category	Not stratified originally		[18,30]	112 (22.5)
			[31,48]	168 (33.7)
			[49,64]	138 (27.7)
			[65,80]	80 (16.1)
			NA	0 (0)
Education	< 9 years in school	7 (1.41)	⩽ 9 years	45 (9.04)
	Elementary (9 years)	38 (7.63)	9–12 years	239 (48)
	High school (12 years)	239 (48)	>12 years	213 (42.8)
	University/vocational	213 (42.8)		
	NA	1 (0.201)	NA	1 (0.201)
Monthly income	< 30k SEK	280 (56.2)	< 30k SEK	280 (56.2)
	30k–50k SEK	155 (31.1)	⩾ 30k SEK	182 (36.5)
	> 50k SEK	27 (5.42)		
	NA	36 (7.23)	NA	36 (7.23)
Living situation^a^	Alone	139 (0.279)	Alone	139 (27.9)
	With partner	275 (0.552)	Not alone	355 (71.3)
	With child	124 (0.249)		
	With relatives	37 (0.074)		
	Other	12 (0.024)		
	NA	4 (0.008)	NA	4 (0.803)
Housing	Rent	178 (35.7)	Rent	199 (40)
	Owns house	168 (33.7)	Own	256 (51.4)
	Owns apartment	88 (17.7)	Other	36 (7.23)
	Homeless	3 (0.602)		
	Rent sublease	21 (4.22)		
	Other	33 (6.63)		
	NA	7 (1.41)	NA	7 (1.41)
Municipality	< 20k residents	74 (14.9)	No further stratification	
	20k–100k residents	161 (32.3)		
	> 100k residents	125 (25.1)		
	Big city	138 (27.7)		
	NA	0 (0)		
Region name^b^	East Middle Sweden (South)	84 (16.9)	North	93 (18.7)
	Middle Norrland (North)	20 (4.02)	South	403 (80.9)
	North Middle Sweden (North)	43 (8.63)		
	Småland and the islands (South)	44 (8.84)		
	South Sweden (South)	77 (15.5)		
	Stockholm (South)	104 (20.9)		
	Upper Norrland (North)	30 (6.02)		
	West Sweden (South)	94 (18.9)		
	NA	2 (0.402)	NA	2 (0.402)
Place of birth	Sweden	427 (85.7)	Sweden	427 (85.7)
	Africa	4 (0.803)	Other Europe	34 (6.83)
	Asia	20 (4.02)	Outside Europe	37 (7.43)
	Other Europe	34 (6.83)		
	Rest of the world	13 (2.61)		
	NA	0 (0)	NA	0 (0)
Total		498		

NA corresponds to optout answers, except for the region name data that originated form the panel.

a Participants could select multiple answers for living situation.

b The region name is followed by the north/south categorization for the region in round brackets.

### Statistical methods

To explore the perceived change in capabilities, we plotted answer distributions overall and per dimension. To assess how dimensions fared in terms of perceived changes, we calculated the difference between positive and negative perceived change proportions for each dimension, called *balance*:

$$Balance=P_{positive}-P_{negative}$$

To calculate uncertainty for the balance and answer frequencies, and to safeguard against potentially skewed distributions, we used bootstrapping with bias corrected accelerated 95% confidence intervals (CI) [16].

To assess whether perceived changes in capabilities were associated with sociodemographic background variables, we used mixed effects logistic regressions. We included varying intercepts for individuals and fixed intercepts per dimension because each participant contributed 10 observations. As outcome, we dichotomized the Likert scale answers, aggregated over all dimensions. ‘Equal’, ‘higher’, and ‘much higher’ were assigned 0 (positive change), and ‘less’, and ‘much less’, 1 (negative change). We grouped ‘equal’ together with ‘higher’ and ‘much higher’ since we focused on negative changes. Independent variables were the self-reported sociodemographic data and the panel-provided residence region.

First, we calculated variable-specific regressions for each background characteristic. We also conducted multivariable logistic regressions with all background variables included to show a broader picture of the associations and adjust for confounders. To investigate age-group specific effects, we categorized age into 18–30, 31–48, 49–64, 65–80. Further, we grouped education into < 9 years, 9–12 years, and > 12 years; monthly income into < 30,000 SEK per month and > 30,000 per month; living status into living alone or not; housing into renting, owning, or other; region of residence into northern and southern Sweden; and place of birth into Sweden, other European country and rest of the world (Table I).

We excluded optout answers and categories with low counts (< 10) in the variable-specific regressions and any observation including such answers for the multivariable regressions. We assessed collinearity using the variance inflation factor [17]. We conducted all analyses with R version 4.0 [21] and used the boot [18], CAR [19] and lme4 [20] packages for bootstrapping, variance inflation factor calculation and mixed effects logistic regression respectively.

## Results

### Participants’ characteristics

The characteristics of the participants, with a mean age 46.03 years (SD 16.29, range 18–80), are displayed in Table I. Region, age groups and gender proportions in the sample were mostly representative of the population. High school and university education groups were slightly overrepresented. Supplemental Section 6 contains a representativity analysis.

### Self-experienced capability levels

**Figure 1.**
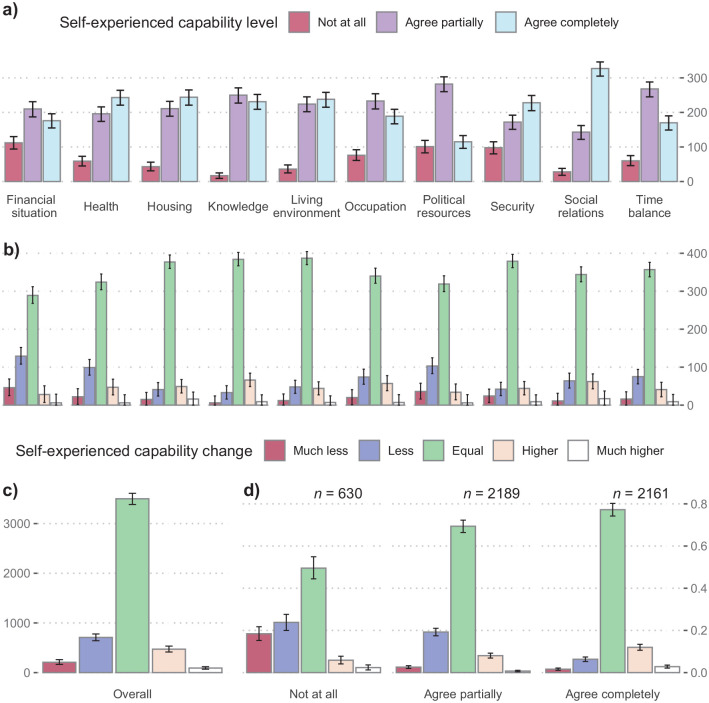
Distribution of survey answers. (a) Answer distribution for self-experienced capability levels per dimension and bootstrapped 95% confidence intervals. (b) Perceived changes per capability dimension during 2020 until the point of data collection between 22 June and 6 July. The dimension is denoted by the label under (a), and possible answers were ‘much less’, ‘less’, ‘equal’, ‘higher’ and ‘much higher’. (c) Distribution of perceived changes added over all dimensions. (d) Perceived changes stratified by the corresponding self-experienced capability level in the respective dimension.

Figure 1(a) shows the current self-experienced capability levels for the 10 dimensions. Whereas most participants answered with ‘agree completely’ for health, living environment, housing, security and social relations, the other dimensions (time balance, financial situation, political resources, knowledge and occupation) had ‘agree partially’ as the most frequent answer. Political resources had most ‘not at all’ answers, followed by financial situation.

### Perceived changes answer distribution

Overall, more respondents answered with ‘much less’ and ‘less’ compared to ‘much higher’ and ‘higher’, although most answers indicated ‘equal’ (Figure 1(c)).

Proportions of perceived negative capability changes were highest for financial situation, followed by health and political resources. Positive change proportions were largest for social relations, knowledge, housing and occupation. ‘Equal’ remained the most frequent answer when stratified per dimension (Figure 1(b)).

When stratifying perceived changes according to the level of self-experienced capability in the according dimension, capabilities among participants who reported ‘not at all’ levels had higher proportions of ‘much less’ and ‘less’ and lower proportions of ‘equal’, ‘higher’ and ‘much higher’ answers compared to participants reporting ‘agree partially’ or ‘agree completely’ (Figure 1(d)).

### Balance of proportion differences

**Figure 2.**
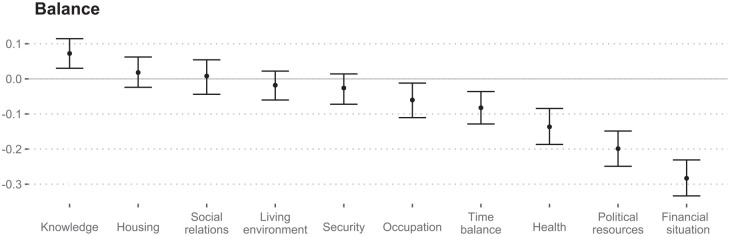
Balance of proportion differences and bootstrapped 95% confidence intervals. Balance is the difference in positive (‘much higher’ and ‘higher’) and negative change proportions (‘much less’ and ‘less’) per dimension.

The balance of positive and negative change proportions indicated that changes were perceived most negatively for financial situation dimension, followed by political resources and health. Time balance and occupation also showed slightly more negative than positive changes. The balance for housing, social relations, living environment and security did not differ significantly from 0. The balance for knowledge was significantly positive (Figure 2).

### Logistic regression

#### Variable-specific regressions

For the logistic regressions, coefficients > 1 indicate higher odds for perceived negative changes and coefficients < 1 lower odds for perceived negative changes. The variable-specific models per background variable indicated that being in the age category of 49–64 and 65–80 compared to being younger than 31; income ⩾ 30,000 SEK compared to < 30,000 SEK per month; and owning housing compared to renting housing were associated with lower odds for a perceived negative change. Being born outside of Europe compared to being born in Sweden and living in the south compared to the north were associated with higher odds of reporting perceived negative changes in capability (Table II).

**Table II.** Odds ratios from variable-specific logistic regressions with one background variable, individual varying intercept and fixed effect per dimension. One model per stratification variable, with 95% confidence intervals (CI). Coefficients with a CI not covering 1 are bold. Reference categories for background variables shown on top. The outcome is the perceived change in capability, aggregated over all dimensions, and dichotomized into negative (‘much less’, ‘less’, outcome 1) and positive (‘equal’, ‘higher’, much higher’, outcome 0) changes. *n* is the number of observations in the model (10 per individual). Fixed effects per dimension not shown; full models available in Supplemental Section 5.

Variable	Category	OR	95.00% CI	*n*
Gender	Men (ref)	1.00		4870
	Woman	1.12	(0.83,1.52)	
Age	[18,30] (ref)	1.00		4980
	[31,48]	0.68	(0.46,1.01)	
	[49,64]	**0.57**	**(0.38,0.87)**	
	[65,80]	**0.52**	**(0.32,0.85)**	
Education	< 12 years (ref)	1.00		4970
	9–12 years	0.67	(0.40,1.14)	
	> 12 years	0.60	(0.35,1.01)	
Income	< 30k SEK (ref)	1.00		4620
	⩾ 30k SEK	**0.58**	**(0.42,0.80)**	
Living situation	Alone (ref)	1.00		4940
	Not alone	0.86	(0.61,1.20)	
Housing	Rent (ref)	1.00		4910
	Own	**0.51**	**(0.37,0.69)**	
	Other	1.35	(0.77,2.36)	
Municipality size	< 20k residents (ref)	1.00		4980
	20k–100k residents	0.76	(0.48,1.22)	
	> 100k residents	0.88	(0.54,1.44)	
	Big city	0.95	(0.59,1.53)	
Region	North (ref)	1.00		4960
	South	**1.60**	**(1.08,2.38)**	
Place of birth	Sweden (ref)	1.00		4980
	Other Europe	1.29	(0.72,2.30)	
	Outside Europe	**2.22**	**(1.29,3.81)**	

#### Multivariable

The odds ratio (OR) for the monthly income category ⩾ 30,000 compared to the baseline of < 30,000 SEK indicated significantly lower odds for stating a negative change. Also, living in a municipality sized between 20,000 and 100,000, compared to a municipality with < 20,000 inhabitants, was significantly associated with lower odds for perceiving a negative capability change. Being born outside Europe compared to Sweden was significantly associated with higher odds of perceived negative change, as was living in the south of Sweden compared to the north and renting compared to owning housing (Table III). The variance inflation factor was below 2 for all variables in all models, indicating that collinearity is not a concern.

**Table III.** Multivariable logistic regression with 95% confidence intervals (CI), with individual varying intercept and fixed effect per dimension. Coefficients with a CI not covering 1 are bold. The outcome is the perceived change in capability, aggregated over all dimensions, and dichotomized into negative (‘much less’, ‘less’, outcome 1) and positive (‘equal’, ‘higher’, much higher’, outcome 0) changes. The first category for every variable is the reference category that the coefficients compare to. *n* is the number of observations in the model (10 per individual). Fixed effects per dimension not shown; full model available in Supplemental Section 5.

Variable	Category	OR	95% CI
Gender	Man (ref)	1	
	Woman	0.94	(0.69,1.29)
Age	[18,30](ref)	1	
	[31,48]	1.06	(0.69,1.62)
	[49,64]	1.00	(0.63,1.58)
	[65,80]	0.87	(0.51,1.50)
Education	⩽ 9 years (ref)	1	
	9–12 years	0.86	(0.48,1.55)
	> 12 years	0.92	(0.50,1.68)
Income	< 30k SEK (ref)	1	
	⩾ 30k SEK	**0.63**	**(0.44,0.90)**
Living status	Alone (ref)	1	
	Not alone	0.95	(0.67,1.36)
Housing	Rent (ref)	1	
	Own	**0.65**	**(0.45,0.93)**
	Other	1.46	(0.76,2.79)
Municipality	< 20k residents (ref)	1	
	20k–100k residents	**0.61**	**(0.38,0.98)**
	> 100k residents	0.77	(0.47,1.25)
	Big city	0.75	(0.45,1.25)
Region	North (ref)	1	
	South	**1.68**	**(1.11,2.55)**
Place of birth	Sweden (ref)	1	
	Other Europe	1.32	(0.72,2.40)
	Outside Europe	**1.86**	**(1.03,3.35)**
	*n*	4460	

To see how results from the aggregated regression would extend to single dimensions, we run fixed effects multivariate regressions also per dimension. Higher income was protective of stating a negative change in all dimensions, except for time balance, and significantly for financial situation, occupation and housing. Owning housing was associated with significantly lower odds for health, housing and time balance. Living in larger municipalities and cities was associated with significantly lower odds for stating negative change in security but seemed to increase odds for negative change in the occupation dimension.

Living in the south was significantly associated with higher odds for negative change for financial situation and time balance. Being born in another European country or outside of Europe was generally associated with higher odds for stating a negative change. Being born outside of Europe increased the odds for negative change strongly and significantly for security and political resources. Dimension-specific multivariable regression results are reported in Supplemental Section 3.

## Discussion

In this study, using recent data, we show for the first COVID-19 wave in 2020 that participants stated more perceived negative than positive capability changes, and a higher proportion stated negative perceived changes if they also stated having low capability in the same capability dimension.

In the capabilities related to financial situation, political resources and health, the proportions of perceived negative changes were highest. Odds for perceived negative change compared to no or positive change were increased for incomes below 30,000 SEK, living in small municipalities in contrast to medium-sized municipalities, being born outside Europe, living in the south of Sweden, and renting instead of owning housing.

### Quality-of-life consequences

Participants perceived that their financial situation was the most negatively changed capability. Statistics from the first half of 2020 on the gross national product [22], layoffs, notices and unemployment [23] and a jump of social insurance allowances in April [24] support this result. Sweden is highly dependent on international trade [25], and hence pessimistic news about the global economy may also have influenced the perception of a more vulnerable financial situation.

Another capability with negative change was political resources. People’s trust in the government, authorities and public media varied during the first four months of the pandemic, but the trust in the Public Health Agency of Sweden (PHAS) has been around 70% and in the healthcare sector around 80%, which is quite high in the Swedish context [26–28]. The negative perception is perhaps a sign of a more general pessimism regarding the possibility to manage and influence one’s own and relatives’ life circumstances during the COVID-19 pandemic.

The indication of a negative change in the capability of heath is reasonable since it could refer to a broader health perception, involve worries and suffering from the COVID-19 virus, and consequences linked to healthcare supply and demand. However, results from a web survey by the PHAS in May 2020 indicate that self-reported mental health and health-related behaviours such as alcohol consumption, smoking, eating habits and physical activity are similar to levels before the pandemic [29].

The capability of knowledge enough to work with or engage in interesting issues and purposeful activities showed a slight positive change. The perception of increased knowledge could depend on changes in the view of interesting or purposeful activities and on more available time for self-improvement. Another possibility is that perceived changes in capability are prone to short-term seasonal effects, similarly to those found for health-related quality of life [30].

The socioeconomic indicators of low income, being born outside Europe and renting housing were more associated with perceived loss of capabilities than medium–high income, being born in Sweden and owning housing. These results are reasonable since the pandemic is likely to disproportionally affect persons in weaker socioeconomic positions [4]. A perceived loss of capability was also more common among participants living in a southern compared to a northern region, and in small compared to medium–big-sized municipalities.

The results from the dimension-specific multivariable regression indicate that the municipality size effect may be mostly related to the security dimension. Other dimension-specific effects were plausible, such as the protective effect of higher income for the capability of financial situation, or the protective effect of owning housing for the housing dimension. Being born outside of Europe was strongly and significantly related to perceived negative change in security and political resources. Living in the south was related to stating a negative change in the financial situation or time balance dimension. Understanding the geographical inequality of perceived capability loss is complex due to large heterogeneity and needs further investigation. Reasons for increased perception of negative change in southern regions may be connected to degree of urbanization, education and income levels, population share with migration background, and COVID-19 spread patterns.

Other research efforts have found negative effects on general quality of life connected to the COVID-19 pandemic. Another Swedish study used repeated measurements of a visual analogue scale and EQ-5D and found a decrease in quality of life [31]. Results in an Indian context indicated that quality of life is related to COVID-19 anxiety [32]. Further, it has been reported from China that the pandemic caused a mild stressful impact [33] and negative impacts on quality of life connected to worrying about getting COVID-19 [34].

### Strengths and limitations

Our study has several limitations. Most apparent is the cross-sectional study design relying on self-reported perceived changes that may be prone to recall bias. We did not conduct prior sample size calculations, and the sample size may have been low for the multivariate regression. We also did not validate or pre-test the questions on perceived negative changes, and the stratified web panel sample may not have been representative. Due to the bias towards higher education, negative changes could have been underestimated in the balance analysis given that higher education reduced odds for perceived negative capability change, albeit not significantly.

The main strengths include the use of a broad framework that allows to compare different quality-of-life dimensions and up-to-date data, linked to the large-scale occurrence of a new pandemic.

The indirect consequences of the COVID-19 pandemic on health and quality of life may vary between countries due to different preventative measures undertaken. This study was performed in Sweden, with a strategy mostly based on voluntary change during the spring of 2020 [2]. Hence, our results should be generalized with caution. Nevertheless, countries are economically and culturally interconnected, and some of the results may be valid for other settings.

## Conclusions

We found self-reported negative capability changes for financial situation, health and political resources in Sweden during the first wave of the COVID-19 pandemic in spring 2020. For the majority of capability dimensions – namely, housing, knowledge, living environment, occupation, security, social relations and time balance – we measured limited or slightly positive perceived change.

Negative and unequal quality-of-life consequences where vulnerable groups in weaker socioeconomic positions are affected harder by the pandemic should be reflected by mitigation and relief policies.

Moreover, the results raise questions regarding capability change over time and the pandemic’s long-term quality of life effects that should be addressed by longitudinal research.

## Supplemental Material

sj-docx-1-sjp-10.1177_14034948211023633 – Supplemental material for Perceived changes in capability during the COVID-19 pandemic: A Swedish cross-sectional study from June 2020 Click here for additional data file. Supplemental material, sj-docx-1-sjp-10.1177_14034948211023633 for Perceived changes in capability during the COVID-19 pandemic: A Swedish cross-sectional study from June 2020 by Kaspar Walter Meili, Håkan Jonsson, Lars Lindholm and Anna Månsdotter in Scandinavian Journal of Public Health
