# Supplementary material for: Perceived changes in capability during the COVID-19 pandemic: A Swedish cross-sectional study from June 2020
Source: Scand J Public Health. 2021 Jul 2;50(1):102–10. doi: 10.1177/14034948211023633 (PMC8808229; doi:10.1177/14034948211023633)
Supplement: sj-docx-1-sjp-10.1177_14034948211023633 – Supplemental material for Perceived changes in capability during the COVID-19 pandemic: A Swedish cross-sectional study from June 2020 [file sj-docx-1-sjp-10.1177_14034948211023633.docx]

# Supplemental Material

For
*Perceived changes in capability during the COVID-19 pandemic: A Swedish cross-sectional study from June 2020*

Contents

[Supplemental Material 1](#_Toc67435063)

[Section 1: Survey screenshots 3](#_Toc67435064)

[Section 2: Survey questions and translations 7](#_Toc67435065)

[Statements 7](#_Toc67435066)

[Sociodemographic background questions 8](#_Toc67435067)

[Section 3: Multivariable logistic regression stratified per dimension 10](#_Toc67435068)

[Section 4: Full results variable-specific logistic regression 12](#_Toc67435069)

[Section 5: Full results multivariable logistic regression 14](#_Toc67435070)

[Section 6: Survey sample representativity 16](#_Toc67435071)

[Age analysis stratification 17](#_Toc67435072)

[Age panel stratification 18](#_Toc67435073)

[Region 19](#_Toc67435074)

[Education 21](#_Toc67435075)

Authors

Kaspar Walter Meili,
Department of Epidemiology and Global Health, Umeå University

Håkan Jonsson, Department of Epidemiology and Global Health,
Umeå University

Lars Lindholm, Department of Epidemiology and Global Health,
Umeå University

Anna Månsdotter, Department of Epidemiology and Global Health,
Umeå University

# Section 1: Survey screenshots


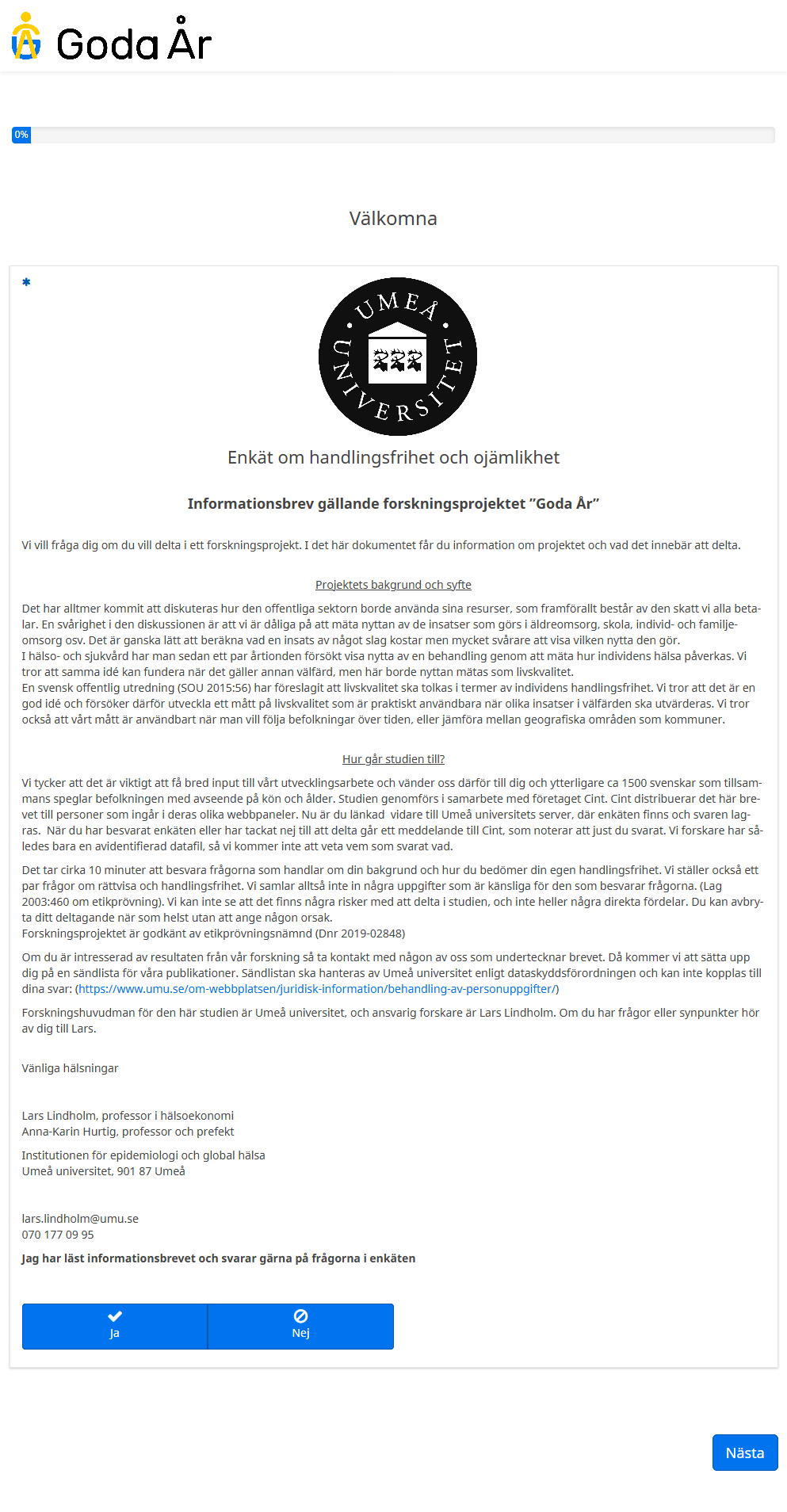


Page 1
Figure S1


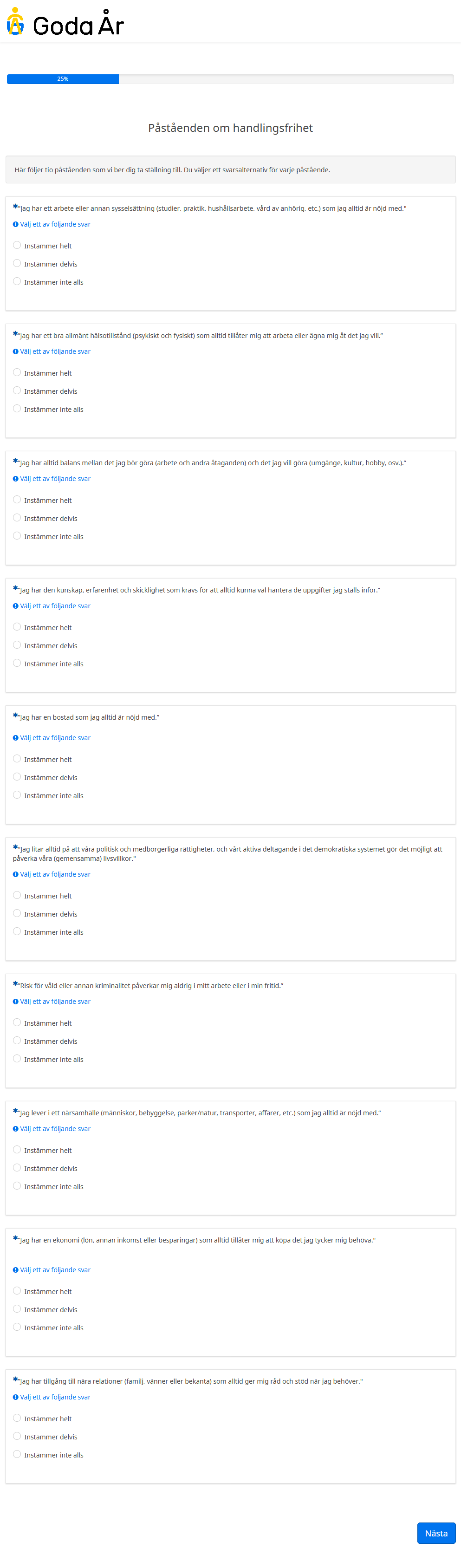
Page 2
Figure S2

Page 3
Figure S3


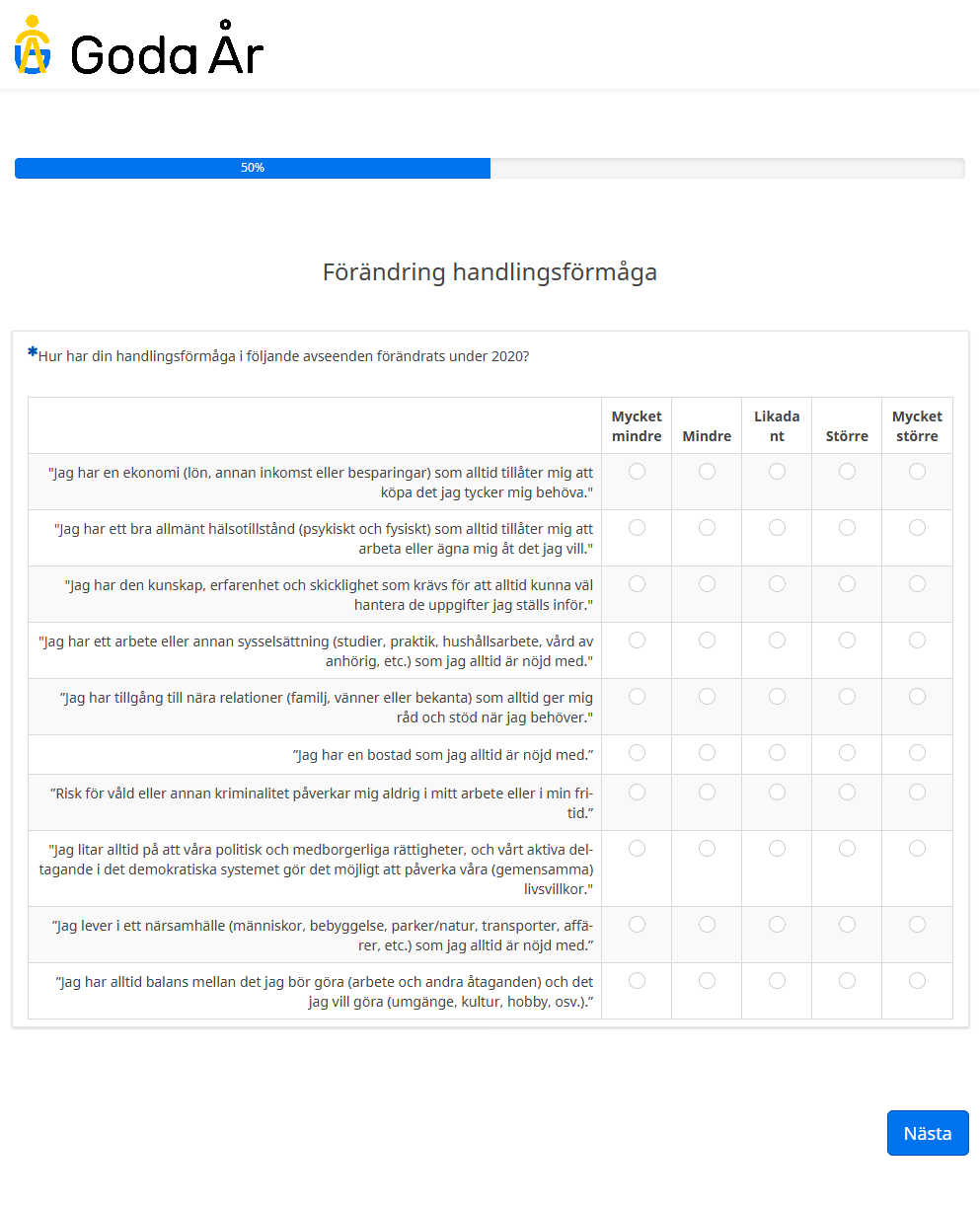


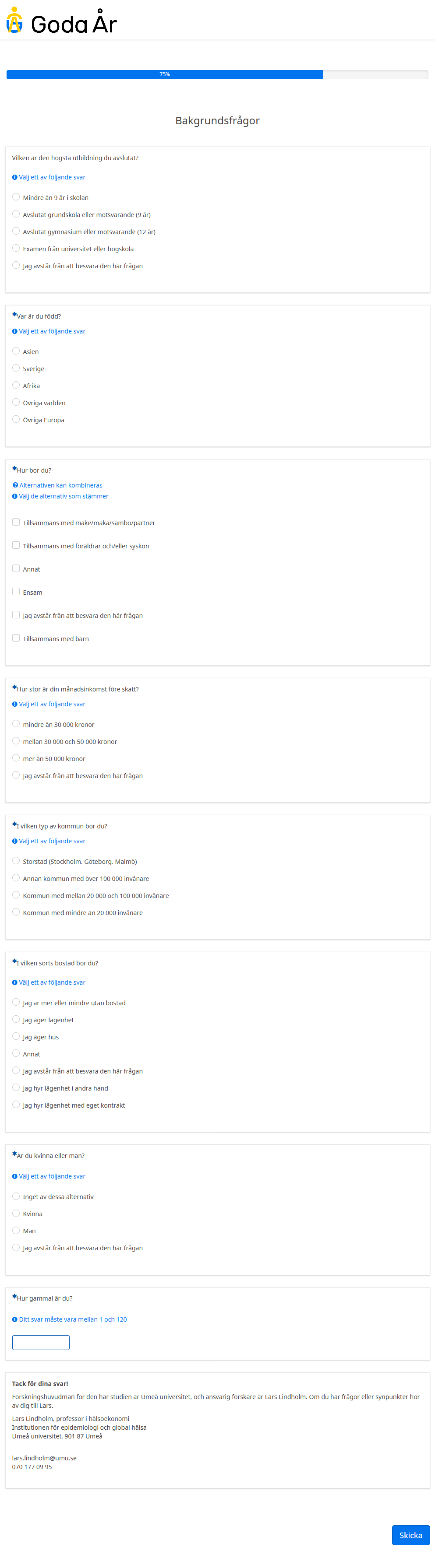
Page 4
Figure S4

# Section 2: Survey questions and translations

## Statements

The statements appeared on page 2 and 3 of the survey. On page 2 participants stated for each statement their baseline capability. On page 3 they stated for each statement the perceived change in capability on a 5-item Likert scale.

| **Question Swedish** | **Question English** |
| --- | --- |
| *Här följer tio påståenden som vi ber dig ta ställning till. Du väljer ett svarsalternativ för varje påstående.*   - Instämmer helt - Instämmer delvis - Instämmer inte alls | *Here follow ten statements that we ask you to answer.*   - Agree completely - Agree partially - Not agree at all |
| *Hur har din handlingsförmåga i följande avseenden förändrats under 2020?*   - Mycket mindre - Mindre - Likadant - Större - Mycket större | *How did your capability change in respect to the following statements during 2020?*   - Much less - Less - Equal - Higher - Much higher |

Table S1. Answer translated answer options for self-rated capability levels and perceived capability change.

| **Capability** | **Swedish** | **English** |
| --- | --- | --- |
| *Financial situation* | ”Jag har en ekonomi (lön, annan inkomst eller besparingar) som alltid tillåter mig att köpa det jag tycker mig behöva." | “I have a financial situation (salary, other income, or saving) that always allows me to buy what I think I need” |
| *Health* | ”Jag har ett bra allmänt hälsotillstånd (psykiskt och fysiskt) som alltid tillåter mig att arbeta eller ägna mig åt det jag vill.” | ”I have good general health (physical and mental) that allows me to work or to do what I want” |
| *Knowledge* | ”Jag har den kunskap, erfarenhet och skicklighet som krävs för att alltid kunna väl hantera de uppgifter jag ställs inför.” | ”I have the knowledge, experience, and skills that are needed to always handle that tasks ahead of me”. |
| *Occupation* | "Jag har ett arbete eller annan sysselsättning (studier, praktik, hushållsarbete, vård av anhörig, etc.) som jag alltid är nöjd med." | ”I have work or another occupation (studies, internship, household work, care of relatives etc. ) that I am always satisfied with”. |
| *Social relations* | ”Jag har tillgång till nära relationer (familj, vänner eller bekanta) som alltid ger mig råd och stöd när jag behöver." | ”I have access to close relations (family, friends or acquaintances) that always give me advice and support when I need it”. |
| *Housing* | ”Jag har en bostad som jag alltid är nöjd med.” | ”I have housing that I am always satisfied with”. |
| *Security* | ”Risk för våld eller annan kriminalitet påverkar mig aldrig i mitt arbete eller i min fritid.” | ”Risk for violence or other crime does never affect me during my work or my leisure time”. |
| Time balance | ”Jag har alltid balans mellan det jag bör göra (arbete och andra åtaganden) och det jag vill göra (umgänge, kultur, hobby, osv.).” | ”I always have a balance between what I should do and want to do (socializing, culture, hoppy, etc. )”. |
| Living environment | ”Jag lever i ett närsamhälle (människor, bebyggelse, parker/natur, transporter, affärer, etc.) som jag alltid är nöjd med.” | ”I live in an environment (people, buildings, nature, transport, etc. ) that I am always satisfied with”. |
| Political and civil rights | "Jag litar alltid på att våra politisk och medborgerliga rättigheter, och vårt aktiva deltagande i det demokratiska systemet gör det möjligt att påverka våra (gemensamma) livsvillkor." | ”I trust always that our political and civil rights, and our active participation in the democratic system make it possible to change our (common) living conditions”. |

Table S2. Translated capability statements.

## Sociodemographic background questions

Appeared on page 4.

| **Question Swedish** | **Question English** |
| --- | --- |
| *Vilken är den högsta utbildning du avslutat?*   - Mindre än 9 år i skolan - Avslutat grundskola eller motsvarande (9 år) - Avslutat gymnasium eller motsvarande (12 år) - Examen från universitet eller högskola - Jag avstår från att besvara den här frågan | What is your highest education that you finished?   - Less than 9 years in the school - Finished basic schooling or similar (9 years) - Finished high school or similar (12 years) - Degree from university or vocal university - I abstain from answering this question |
|  |  |
| *Är du kvinna eller man?*   - Jag avstår från att besvara den här frågan - Kvinna - Inget av dessa alternativ - Man | *Are you man or woman?*   - I abstain from answering this question - Woman - None of those alternatives - Man |
|  |  |
| *Var är du född?*   - Asien - Övriga världen - Övriga Europa - Afrika - Sverige | *Where are you born?*   - Asia - Rest of the world - Rest of Europe - Africa - Sweden |
|  |  |
| *Hur gammal är du?* | *How old are you?* |
|  |  |
| *Hur stor är din månadsinkomst före skatt?*   - mindre än 30 000 kronor - mellan 30 000 och 50 000 kronor - mer än 50 000 kronor - Jag avstår från att besvara den här frågan | *How big is your monthly income before taxes?*   - Less than 30 000 krona - Between 30 000 and 50 000 krona - More than 50 000 krona - I abstain from answering this question |
|  |  |
| *I vilken sorts bostad bor du?*   - Jag hyr lägenhet i andra hand - Annat - Jag äger hus - Jag hyr lägenhet med eget kontrakt - Jag är mer eller mindre utan bostad - Jag äger lägenhet - Jag avstår från att besvara den här frågan | *In what kind of accommodation do you live?*   - I rent an apartment with a sublet contract - Other - I own a house - I rent an apartment - I am more or less without accommodation - I own an apartment - I abstain from answering this question |
|  |  |
| *Hur bor du? (Multiple choice)*   - Tillsammans med barn - Tillsammans med make/maka/sambo/partner - Jag avstår från att besvara den här frågan - Tillsammans med föräldrar och/eller syskon - Annat - Ensam | How do you live? (Multiple choice)   - Together with children - Together with husband/wife/registered partner /partner - I abstain from answering this question - Together with parents and/or siblings - Other - Alone |
|  |  |
| *I vilken typ av kommun bor du?*   - Storstad (Stockholm, Göteborg, Malmö) - Annan kommun med över 100 000 invånare - Kommun med mellan 20 000 och 100 000 invånare - Kommun med mindre än 20 000 invånare | *In what type of community do you live?*   - Big city (Stockholm, Gothenburg, Malmö) - Other municipality with more than 100 000 residents - Municipality between 20 000 and 100 000 residents - Municipality with less than 20 000 residents |

Table S3. Translated sociodemographic background questions.

# Section 3: Multivariable logistic regression stratified per dimension

|  | Financial   situation | Health | Knowledge | Occupation | Social relations | Housing | Security | Political resources | Living environment | Time balance |
| --- | --- | --- | --- | --- | --- | --- | --- | --- | --- | --- |
| *Gender* |  |  |  |  |  |  |  |  |  |  |
| Man (ref) | 1 | 1 | 1 | 1 | 1 | 1 | 1 | 1 | 1 | 1 |
| Woman | 1.10 | 1.34 | 1.19 | 0.82 | 0.95 | 0.53 | 1.07 | 0.74 | 0.70 | 1.15 |
|  | (0.71,1.70) | (0.84,2.16) | (0.57,2.51) | (0.48,1.38) | (0.56,1.61) | (0.27,1.02) | (0.59,1.96) | (0.47,1.15) | (0.37,1.28) | (0.69,1.95) |
| *Age* |  |  |  |  |  |  |  |  |  |  |
| [18,30] (ref) | 1 | 1 | 1 | 1 | 1 | 1 | 1 | 1 | 1 | 1 |
| [31,48] | 0.78 | 1.12 | 2.20 | 1.59 | 1.01 | 0.67 | 1.45 | 1.83 | 1.20 | 0.72 |
|  | (0.44,1.39) | (0.59,2.17) | (0.83,6.48) | (0.80,3.28) | (0.49,2.12) | (0.29,1.52) | (0.65,3.38) | (0.98,3.53) | (0.53,2.89) | (0.36,1.44) |
| [49,64] | 0.76 | 1.33 | 1.82 | 1.29 | 1.28 | 0.87 | 1.28 | 1.21 | 1.14 | 0.95 |
|  | (0.41,1.41) | (0.66,2.69) | (0.61,5.77) | (0.60,2.83) | (0.60,2.77) | (0.35,2.11) | (0.53,3.20) | (0.61,2.44) | (0.47,2.89) | (0.46,1.98) |
| [65,80] | 0.85 | 1.97 | 0.73 | 0.64 | 0.90 | **0.26** | 0.89 | 2.06 | 0.63 | 0.74 |
|  | (0.41,1.75) | (0.89,4.39) | (0.10,3.62) | (0.22,1.72) | (0.34,2.26) | **(0.05,0.92)** | (0.27,2.76) | (0.97,4.46) | (0.19,1.91) | (0.29,1.81) |
| *Education* |  |  |  |  |  |  |  |  |  |  |
| <=9 years (ref) | 1 | 1 | 1 | 1 | 1 | 1 | 1 | 1 | 1 | 1 |
| 9-12 years | 0.81 | 1.16 | 0.84 | 2.42 | 0.53 | 2.19 | 0.68 | 0.72 | 0.64 | 0.82 |
|  | (0.38,1.76) | (0.49,3.00) | (0.27,3.19) | (0.85,8.86) | (0.22,1.33) | (0.66,10.12) | (0.25,2.07) | (0.33,1.63) | (0.23,2.13) | (0.33,2.25) |
| >12 years | 0.59 | 1.24 | 0.60 | 2.75 | 0.69 | 2.86 | 0.75 | 0.64 | 0.81 | 1.14 |
|  | (0.26,1.33) | (0.51,3.29) | (0.17,2.46) | (0.93,10.28) | (0.29,1.79) | (0.81,13.76) | (0.26,2.41) | (0.28,1.50) | (0.28,2.76) | (0.45,3.20) |
| *Income* |  |  |  |  |  |  |  |  |  |  |
| <30k SEK (ref) | 1 | 1 | 1 | 1 | 1 | 1 | 1 | 1 | 1 | 1 |
| >=30k SEK | **0.45** | 0.71 | 0.93 | **0.44** | 0.79 | **0.24** | 0.69 | 0.67 | 0.85 | 1.38 |
|  | **(0.27,0.74)** | (0.41,1.21) | (0.38,2.15) | **(0.23,0.82)** | (0.43,1.45) | **(0.08,0.58)** | (0.34,1.37) | (0.41,1.10) | (0.42,1.66) | (0.78,2.46) |
| *Living status* |  |  |  |  |  |  |  |  |  |  |
| Alone | 1 | 1 | 1 | 1 | 1 | 1 | 1 | 1 | 1 | 1 |
| Not alone | 0.82 | 0.95 | 1.14 | 1.03 | 0.64 | 1.10 | 1.64 | 1.27 | 0.73 | 1.17 |
|  | (0.51,1.33) | (0.56,1.61) | (0.51,2.75) | (0.57,1.88) | (0.36,1.13) | (0.54,2.28) | (0.82,3.48) | (0.77,2.14) | (0.38,1.44) | (0.66,2.13) |
| *Housing* |  |  |  |  |  |  |  |  |  |  |
| Rent (ref) | 1 | 1 | 1 | 1 | 1 | 1 | 1 | 1 | 1 | 1 |
| Own | 0.63 | **0.44** | 0.46 | 0.64 | 0.78 | **0.32** | 0.65 | 1.05 | 1.18 | **0.49** |
|  | (0.39,1.01) | **(0.25,0.76)** | (0.19,1.09) | (0.35,1.15) | (0.43,1.43) | **(0.14,0.70)** | (0.32,1.30) | (0.63,1.74) | (0.60,2.34) | **(0.27,0.89)** |
| Other | 2.30 | 1.22 | 1.89 | **3.55** | 1.32 | 0.76 | 1.37 | 0.54 | 0.30 | 1.20 |
|  | (0.96,5.77) | (0.47,2.98) | (0.53,5.98) | **(1.39,9.08)** | (0.43,3.58) | (0.22,2.23) | (0.44,3.89) | (0.17,1.49) | (0.02,1.62) | (0.42,3.14) |
| *Municipality* |  |  |  |  |  |  |  |  |  |  |
| <20k residents (ref) | 1 | 1 | 1 | 1 | 1 | 1 | 1 | 1 | 1 | 1 |
| 20k-100k residents | 0.86 | 0.52 | 0.80 | 2.10 | 0.63 | 0.65 | **0.31** | 0.62 | 0.48 | 0.53 |
|  | (0.45,1.67) | (0.26,1.05) | (0.29,2.38) | (0.89,5.46) | (0.28,1.41) | (0.25,1.74) | **(0.14,0.69)** | (0.33,1.20) | (0.18,1.28) | (0.25,1.15) |
| >100k residents | 1.06 | 0.65 | 0.81 | **2.51** | 0.79 | 0.64 | **0.26** | 0.72 | 0.72 | 0.57 |
|  | (0.54,2.07) | (0.32,1.32) | (0.27,2.45) | **(1.05,6.56)** | (0.36,1.80) | (0.24,1.78) | **(0.10,0.61)** | (0.37,1.42) | (0.28,1.90) | (0.27,1.25) |
| Big city | 1.15 | 0.78 | 0.62 | 1.43 | 0.88 | 0.45 | **0.32** | 0.67 | 1.12 | 0.47 |
|  | (0.57,2.32) | (0.38,1.62) | (0.18,2.11) | (0.56,3.94) | (0.38,2.06) | (0.15,1.33) | **(0.13,0.76)** | (0.33,1.35) | (0.45,2.95) | (0.21,1.06) |
| *Region* |  |  |  |  |  |  |  |  |  |  |
| North (ref) | 1 | 1 | 1 | 1 | 1 | 1 | 1 | 1 | 1 | 1 |
| South | **2.12** | 1.11 | 0.76 | 1.61 | 1.10 | 1.69 | 1.53 | 1.73 | 1.26 | **2.20** |
|  | **(1.19,3.90)** | (0.61,2.09) | (0.33,1.87) | (0.82,3.37) | (0.56,2.28) | (0.74,4.28) | (0.69,3.68) | (0.96,3.26) | (0.54,3.31) | **(1.06,5.05)** |
| *Place of birth* |  |  |  |  |  |  |  |  |  |  |
| Sweden (ref) | 1 | 1 | 1 | 1 | 1 | 1 | 1 | 1 | 1 | 1 |
| Other Europe | 1.31 | 1.75 | 0.51 | 1.13 | 0.80 | 0.31 | 2.16 | 1.42 | 2.32 | 1.06 |
|  | (0.56,2.93) | (0.74,3.96) | (0.03,2.65) | (0.35,2.99) | (0.22,2.22) | (0.02,1.62) | (0.74,5.57) | (0.61,3.16) | (0.85,5.72) | (0.37,2.65) |
| Outside Europe | 1.34 | 1.22 | 2.18 | 1.16 | 1.74 | 1.76 | **3.06** | **2.98** | 2.01 | 1.13 |
|  | (0.59,3.01) | (0.47,2.89) | (0.65,6.29) | (0.44,2.80) | (0.67,4.18) | (0.61,4.62) | **(1.14,7.62)** | **(1.32,6.68)** | (0.66,5.41) | (0.39,2.83) |
| N | 446 | 446 | 446 | 446 | 446 | 446 | 446 | 446 | 446 | 446 |

Table S4. Multivariable fixed effects logistic regression. Per dimension, with 95% confidence intervals (CI). The outcome is the perceived change in capability dichotomized into negative (“Much less”, “Less”, outcome 1) and positive (“Equal”, ”Higher”, Much higher”, outcome 0) changes. First category for every variable is the reference category that the coefficients compare to (intercept not shown). N is the number of observations in the model.

# Section 4: Full results variable-specific logistic regression

|  | Gender | Age | Education | Income | Living situation | Housing | Municipality size | Region | Place of birth |
| --- | --- | --- | --- | --- | --- | --- | --- | --- | --- |
| *Gender* |  |  |  |  |  |  |  |  |  |
| Man (ref) | 1 |  |  |  |  |  |  |  |  |
|  |  |  |  |  |  |  |  |  |  |
| Woman | 1.12 |  |  |  |  |  |  |  |  |
|  | (0.83,1.52) |  |  |  |  |  |  |  |  |
| *Age* |  |  |  |  |  |  |  |  |  |
| [18-30] (ref) |  | 1 |  |  |  |  |  |  |  |
|  |  |  |  |  |  |  |  |  |  |
| [31,48] |  | 0.68 |  |  |  |  |  |  |  |
|  |  | (0.46,1.01) |  |  |  |  |  |  |  |
| [49,64] |  | **0.57** |  |  |  |  |  |  |  |
|  |  | **(0.38,0.87)** |  |  |  |  |  |  |  |
| [65,80] |  | **0.52** |  |  |  |  |  |  |  |
|  |  | **(0.32,0.85)** |  |  |  |  |  |  |  |
| *Education* |  |  |  |  |  |  |  |  |  |
| < 12 years (ref) |  |  | 1 |  |  |  |  |  |  |
|  |  |  |  |  |  |  |  |  |  |
| 9-12 years |  |  | 0.67 |  |  |  |  |  |  |
|  |  |  | (0.40,1.14) |  |  |  |  |  |  |
| >12 years |  |  | 0.60 |  |  |  |  |  |  |
|  |  |  | (0.35,1.01) |  |  |  |  |  |  |
| *Income* |  |  |  |  |  |  |  |  |  |
| <30k SEK |  |  |  | 1 |  |  |  |  |  |
|  |  |  |  |  |  |  |  |  |  |
| >=30k SEK |  |  |  | **0.58** |  |  |  |  |  |
|  |  |  |  | **(0.42,0.80)** |  |  |  |  |  |
| *Living status* |  |  |  |  |  |  |  |  |  |
| Alone |  |  |  |  | 1 |  |  |  |  |
|  |  |  |  |  |  |  |  |  |  |
| Not alone |  |  |  |  | 0.86 |  |  |  |  |
|  |  |  |  |  | (0.61,1.20) |  |  |  |  |
| *Housing* |  |  |  |  |  |  |  |  |  |
|  |  |  |  |  |  |  |  |  |  |
| Own |  |  |  |  |  | **0.51** |  |  |  |
|  |  |  |  |  |  | **(0.37,0.69)** |  |  |  |
| Other |  |  |  |  |  | 1.35 |  |  |  |
|  |  |  |  |  |  | (0.77,2.36) |  |  |  |
| *Municipality* |  |  |  |  |  |  |  |  |  |
| <20k residents |  |  |  |  |  |  | 1 |  |  |
|  |  |  |  |  |  |  |  |  |  |
| 20k-100k residents |  |  |  |  |  |  | 0.76 |  |  |
|  |  |  |  |  |  |  | (0.48,1.22) |  |  |
| >100k residents |  |  |  |  |  |  | 0.88 |  |  |
|  |  |  |  |  |  |  | (0.54,1.44) |  |  |
| Big city |  |  |  |  |  |  | 0.95 |  |  |
|  |  |  |  |  |  |  | (0.59,1.53) |  |  |
| *Region* |  |  |  |  |  |  |  |  |  |
| North (ref) |  |  |  |  |  |  |  | 1 |  |
|  |  |  |  |  |  |  |  |  |  |
| South |  |  |  |  |  |  |  | **1.60** |  |
|  |  |  |  |  |  |  |  | **(1.08,2.38)** |  |
| *Place of birth* |  |  |  |  |  |  |  |  |  |
| Sweden (ref) |  |  |  |  |  |  |  |  | 1 |
|  |  |  |  |  |  |  |  |  |  |
| Other Europe |  |  |  |  |  |  |  |  | 1.29 |
|  |  |  |  |  |  |  |  |  | (0.72,2.30) |
| Outside Europe |  |  |  |  |  |  |  |  | **2.22** |
|  |  |  |  |  |  |  |  |  | **(1.29,3.81)** |
| Dimension |  |  |  |  |  |  |  |  |  |
| Financial situation (ref) | **1** | **1** | **1** | **1** | **1** | **1** | **1** | **1** | **1** |
| Health | **0.51** | **0.50** | **0.50** | **0.49** | **0.50** | **0.52** | **0.50** | **0.50** | **0.50** |
|  | **(0.37,0.70)** | **(0.36,0.69)** | **(0.37,0.69)** | **(0.35,0.68)** | **(0.37,0.69)** | **(0.38,0.71)** | **(0.37,0.69)** | **(0.36,0.69)** | **(0.37,0.69)** |
| Housing | **0.14** | **0.16** | **0.16** | **0.17** | **0.15** | **0.16** | **0.16** | **0.16** | **0.16** |
|  | **(0.10,0.21)** | **(0.11,0.23)** | **(0.11,0.23)** | **(0.11,0.25)** | **(0.10,0.22)** | **(0.11,0.23)** | **(0.11,0.23)** | **(0.11,0.23)** | **(0.11,0.23)** |
| Knowledge | **0.10** | **0.10** | **0.10** | **0.10** | **0.10** | **0.10** | **0.10** | **0.10** | **0.10** |
|  | **(0.06,0.15)** | **(0.06,0.15)** | **(0.06,0.15)** | **(0.07,0.16)** | **(0.07,0.15)** | **(0.06,0.15)** | **(0.06,0.15)** | **(0.06,0.15)** | **(0.06,0.15)** |
| Living environment | **0.18** | **0.17** | **0.17** | **0.17** | **0.17** | **0.18** | **0.17** | **0.17** | **0.17** |
|  | **(0.12,0.26)** | **(0.12,0.25)** | **(0.12,0.25)** | **(0.12,0.25)** | **(0.12,0.25)** | **(0.12,0.26)** | **(0.12,0.25)** | **(0.12,0.25)** | **(0.12,0.25)** |
| Occupation | **0.34** | **0.33** | **0.33** | **0.32** | **0.34** | **0.34** | **0.33** | **0.33** | **0.33** |
|  | **(0.24,0.47)** | **(0.24,0.46)** | **(0.24,0.46)** | **(0.22,0.45)** | **(0.24,0.47)** | **(0.24,0.48)** | **(0.24,0.46)** | **(0.24,0.46)** | **(0.24,0.46)** |
| Political resources | **0.64** | **0.64** | **0.64** | **0.67** | **0.64** | **0.65** | **0.64** | **0.64** | **0.64** |
|  | **(0.47,0.88)** | **(0.47,0.87)** | **(0.47,0.87)** | **(0.48,0.92)** | **(0.47,0.88)** | **(0.48,0.89)** | **(0.47,0.87)** | **(0.47,0.87)** | **(0.47,0.87)** |
| Security | **0.19** | **0.20** | **0.20** | **0.19** | **0.20** | **0.20** | **0.20** | **0.20** | **0.20** |
|  | **(0.13,0.28)** | **(0.14,0.28)** | **(0.14,0.28)** | **(0.13,0.28)** | **(0.14,0.29)** | **(0.14,0.29)** | **(0.14,0.28)** | **(0.14,0.28)** | **(0.14,0.28)** |
| Social relations | **0.25** | **0.24** | **0.24** | **0.26** | **0.24** | **0.24** | **0.24** | **0.24** | **0.24** |
|  | **(0.18,0.36)** | **(0.17,0.34)** | **(0.17,0.34)** | **(0.18,0.38)** | **(0.17,0.35)** | **(0.17,0.34)** | **(0.17,0.34)** | **(0.17,0.34)** | **(0.17,0.34)** |
| Time balance | **0.30** | **0.32** | **0.32** | **0.32** | **0.31** | **0.32** | **0.32** | **0.32** | **0.32** |
|  | **(0.22,0.43)** | **(0.23,0.44)** | **(0.23,0.44)** | **(0.22,0.45)** | **(0.22,0.44)** | **(0.23,0.45)** | **(0.23,0.44)** | **(0.23,0.44)** | **(0.23,0.44)** |
| N | 4870.00 | 4980.00 | 4970.00 | 4620.00 | 4940.00 | 4910.00 | 4980.00 | 4960.00 | 4980.00 |

Table S5. Full results variable-specific logistic regression per background variable. One model per column. With 95% confidence intervals (CI). The outcome is the perceived change in capability dichotomized into negative (“Much less”, “Less”, outcome 1) and positive (“Equal”, ”Higher”, Much higher”, outcome 0) changes. First category for every variable is the reference category that the coefficients compare to (intercept not shown). N is the number of observations in the model.

# Section 5: Full results multivariable logistic regression

| **Variable** | **Category** | **OR** | **95% CI** |
| --- | --- | --- | --- |
| *Gender* | Man (ref) | 1 |  |
|  | Woman | 0.94 | (0.69,1.29) |
| *Age* | [18,30 (ref)] | 1 |  |
|  | [31,48] | 1.06 | (0.69,1.62) |
|  | [49,64] | 1.00 | (0.63,1.58) |
|  | [65,80] | 0.87 | (0.51,1.50) |
| *Education* | <=9 years (ref) | 1 |  |
|  | 9-12 years | 0.86 | (0.48,1.55) |
|  | >12 years | 0.92 | (0.50,1.68) |
| *Income* | <30k SEK (ref) | 1 |  |
|  | >=30k SEK | 0.63 | **(0.44,0.90)** |
| *Living status* | Alone (ref) | 1 |  |
|  | Not alone | 0.95 | (0.67,1.36) |
| *Housing* | Rent (ref) | 1 |  |
|  | Own | 0.65 | **(0.45,0.93)** |
|  | Other | 1.46 | (0.76,2.79) |
| *Municipality* | <20k residents (ref) | 1 |  |
|  | 20k-100k residents | 0.61 | **(0.38,0.98)** |
|  | >100k residents | 0.77 | (0.47,1.25) |
|  | Big city | 0.75 | (0.45,1.25) |
| *Region* | North (ref) | 1 |  |
|  | South | 1.68 | **(1.11,2.55)** |
| *Place of birth* | Sweden (ref) | 1 |  |
|  | Other Europe | 1.32 | (0.72,2.40) |
|  | Outside Europe | 1.86 | **(1.03,3.35)** |
| *Dimension* | Financial  situation (ref) | 1 |  |
|  | *Health* | *0.50* | ***(0.35,0.70)*** |
|  | Housing | 0.15 | **(0.10,0.23)** |
|  | Knowledge | 0.10 | **(0.07,0.16)** |
|  | Living environment | 0.18 | **(0.12,0.27)** |
|  | Occupation | 0.33 | **(0.23,0.47)** |
|  | Political resources | 0.69 | **(0.49,0.95)** |
|  | Security | 0.20 | **(0.13,0.29)** |
|  | Social relations | 0.29 | **(0.20,0.41)** |
|  | Time balance | 0.32 | **(0.22,0.46)** |
|  | N | 4460 |  |

Table S6. Full result multivariable mixed effects logistic regression. With individual varying intercept and fixed effect per dimension. With 95% confidence intervals (CI). The outcome is the perceived change in capability dichotomized into negative (“Much less”, “Less”, outcome 1) and positive (“Equal”, ”Higher”, Much higher”, outcome 0) changes. First category for every variable is the reference category that the coefficients compare to (intercept not shown). N is the number of observations in the model.

# Section 6: Survey sample representativity

Data for age, gender, and region of residence obtained from Statistic Sweden for 2019 (Table Population after region, civil status, age and sex [Folkmängden efter region, civilstånd, ålder och kön. År 1968 – 2020], <https://www.statistikdatabasen.scb.se/sq/104054>), with NUTS2 region variable selected. Ages < 18 and ages > 80 excluded.

Data for education obtained from Statistics Sweden for 2019 (Table Population 16-95+ years of age by region, level of education, age and year, <http://www.statistikdatabasen.scb.se/sq/104062>). Ages < 18 and ages > 80 excluded.

Panel census stratification target (n=500)

| **Gender** | **N** | **Percent** |
| --- | --- | --- |
| Men | 250 | 50.00 |
| Women | 250 | 50.00 |

Survey sample

| **Gender** | **N** | **Percent** |
| --- | --- | --- |
| Men | 242 | 48.59 |
| Women | 245 | 49.20 |
| Other | 7 | 1.41 |
| Optout | 4 | 0.80 |

SCB data

| **Gender** | **N** | **Percent** |
| --- | --- | --- |
| Men | 3887700 | 50.65 |
| Women | 3788377 | 49.35 |

Chi-squared goodness-of-fit survey sample proportions to SCB population proportions: X^2^: 0.18, pval: 0.67

Null hypothesis of equal proportions not rejected, support for representativity

(Other and Optout dropped for Chi-squared test)

## Age analysis stratification

(The stratification used in the main article)

Survey sample analysis stratification

| **Age group** | **N** | **Percent** |
| --- | --- | --- |
| [18,30] | 112 | 22.49 |
| [31,48] | 168 | 33.73 |
| [49,64] | 138 | 27.71 |
| [65,80] | 80 | 16.06 |

SCB data analyses stratification

| **Age group** | **N** | **Percent** |
| --- | --- | --- |
| [18,30] | 1700479 | 22.15 |
| (30,48] | 2384503 | 31.06 |
| (48,64] | 1996732 | 26.01 |
| (64,80] | 1594363 | 20.77 |

Chi-squared goodness-of-fit survey sample proportions to SCB population proportions: X^2^: X^2^: 7.03, pval: 0.07

Null hypothesis of equal proportions not rejected, support for representativity

## Age panel stratification

(The stratification used for the stratified sampling)

Panel census stratification target (n=500)

| **Age group** | **N** | **Percent** |
| --- | --- | --- |
| 18-22 | 45 | 9.00 |
| 23-35 | 115 | 23.00 |
| 36-55 | 175 | 35.00 |
| 56-80 | 165 | 33.00 |

Survey sample panel stratification

| **Age group** | **N** | **Percent** |
| --- | --- | --- |
| [18,22] | 35 | 7.03 |
| (22,35] | 124 | 24.90 |
| (35,55] | 182 | 36.55 |
| (55,80] | 157 | 31.53 |

SCB data panel stratification

| **Age group** | **N** | **Percent** |
| --- | --- | --- |
| [18,22] | 565262 | 7.36 |
| (22,35] | 1833787 | 23.89 |
| (35,55] | 2631971 | 34.29 |
| (55,80] | 2645057 | 34.46 |

Chi-squared goodness-of-fit survey sample proportions to SCB population proportions: X^2^: X^2^: 2.27, pval: 0.52

Null hypothesis of equal proportions not rejected, support for representativity

## Region

Panel census stratification target (n=500)

| **Region** | **N** | **Percent** |
| --- | --- | --- |
| Middle Norrland | 20 | 4.00 |
| North Middle Sweden | 45 | 9.00 |
| Småland med öarna | 45 | 9.00 |
| Stockholm | 105 | 21.00 |
| South Sweden | 75 | 15.00 |
| West Sweden | 95 | 19.00 |
| East Middle Sweden | 85 | 17.00 |
| Upper Norrland | 30 | 6.00 |

Survey sample

| **Region** | **N** | **Percent** |
| --- | --- | --- |
| NA | 2 | 0.40 |
| East Middle Sweden | 84 | 16.87 |
| Middle Norrland | 20 | 4.02 |
| North Middle Sweden | 43 | 8.63 |
| Småland and the islands | 44 | 8.84 |
| South Sweden | 77 | 15.46 |
| Stockholm | 104 | 20.88 |
| Upper Norrland | 30 | 6.02 |
| West Sweden | 94 | 18.88 |

SCB data

| **Region** | **N** | **Percent** |
| --- | --- | --- |
| East Middle Sweden | 1282173 | 16.70 |
| Middle Norrland | 279802 | 3.65 |
| North Middle Sweden | 638729 | 8.32 |
| Småland and the islands | 640347 | 8.34 |
| South Sweden | 1136058 | 14.80 |
| Stockholm | 1774040 | 23.11 |
| Upper Norrland | 393829 | 5.13 |
| West Sweden | 1531099 | 19.95 |

Chi-squared goodness-of-fit survey sample proportions to SCB population proportions: X^2^: 2.68, pval: 0.91

Null hypothesis of equal proportions not rejected, support for representativity

(NA dropped for Chi-squared test)

## Education

Panel census stratification target (n=500)

| **Education** | **N** | **Percent** |
| --- | --- | --- |
| None completed | 5 | 1.00 |
| Secondary School | 35 | 7.00 |
| High School/Tertiary/Tech. College | 215 | 43.00 |
| University/Higher Education | 240 | 48.00 |
| Postgraduate Education | 5 | 1.00 |

Survey sample

| **Education** | **N** | **Percent** |
| --- | --- | --- |
| Optout | 1 | 0.20 |
| <9 years in school | 7 | 1.41 |
| Elementary (9 years) | 38 | 7.63 |
| High school (12 years) | 239 | 47.99 |
| University/vocational | 213 | 42.77 |

SCB data

| **Education** | **N** | **Tentative mapping to SCB** | **Percent** |
| --- | --- | --- | --- |
| primary and secondary education less than 9 years (ISCED97 1) | 402925 | <9.00 years in school | 5.25 |
| primary and secondary education 9-10 years (ISCED97 2) | 792604 | Elementary (9.00 years) | 10.3 |
| upper secondary education, 2 years or less (ISCED97 3C) | 1551059 | High school (12.00 years) | 20.2 |
| upper secondary education 3 years (ISCED97 3A) | 1799801 | High school (12.00 years) | 23.4 |
| post-secondary education, less than 3 years (ISCED97 4+5B) | 1131775 | University/vocational | 14.7 |
| post-secondary education 3 years or more (ISCED97 5A) | 1709645 | University/vocational | 22.3 |
| post-graduate education (ISCED97 6) | 90459 | University/vocational | 1.18 |
| no information about level of educational attainment | 197809 | NA | 2.58 |

SCB data mapped to survey stratification

| **Education** | **N** | **Percent** |
| --- | --- | --- |
| <9 years in school | 402925 | 5.25 |
| Elementary (9 years) | 792604 | 10.33 |
| High school (12 years) | 3350860 | 43.65 |
| University/vocational | 2931879 | 38.20 |
| NA | 197809 | 2.58 |

Chi-squared goodness-of-fit survey sample proportions to SCB population proportions: X^2^: X^2^: 21.58, pval: 0

Null hypothesis of equal proportions rejected, no support for representativity

(Optout and NA dropped for chi-squared test)
